# Reliability of a functional electromechanical dynamometer for a bilateral standing press exercise at intensities of 30% and 60% of isometric peak force in trained women

**DOI:** 10.7717/peerj.21777

**Published:** 2026-10-15

**Authors:** Claudio Cifuentes-Zapata, Oscar Andrades-Ramírez, Luis Romero-Vera, David Ulloa-Díaz, Ángela Rodríguez-Perea, Claudio Carvajal-Parodi, Álvaro Huerta Ojeda, Sergio Araya-Sierralta, Luis Javier Chirosa-Ríos

**Affiliations:** 1 Department of Physical Education and Sport, Faculty of Sport Sciences, University of Granada, Granada, Spain; 2 Núcleo de Investigación en Salud, Actividad Física y Deportes ISAFYD, Escuela de Ciencias de la Actividad Física, Universidad de las Américas, Santiago, Chile; 3 Facultad de Educación y Ciencias Sociales, Universidad Andres Bello, Entrenador Deportivo, Concepción, Chile; 4 Facultad de Salud y Ciencias Sociales, Escuela de Ciencias de la Actividad Física, Universidad de las Américas, Concepción, Chile; 5 Department of Sports Sciences and Physical Conditioning, Universidad Católica de la Santísima Concepción, Concepción, Chile; 6 Department of Physical Education and Sport, Faculty of Physical Activity and Sports Sciences, Universidad de León, León, Spain; 7 Facultad de Ciencias de la Rehabilitación y Calidad de Vida, Escuela de Kinesiología, Universidad San Sebastián, Concepción, Chile; 8 Facultad de Humanidades y Educación, Universidad de Atacama, Copiapó, Chile

**Keywords:** Functional electromechanical dynamometry, Mean muscular force, Isometric peak force, Reliability, Mean velocity, Mean power

## Abstract

**Background:**

Determining the reliability of exercise instruments and protocols in the fields of health and sports performance is of great interest due to their close relationship with monitoring and decision-making in athletes. Studies in trained women are limited, and more evidence is needed on performance variables and the reliability of exercise protocols.

**Objective:**

This study aimed to evaluate the reliability of an exercise protocol for determining mean muscle strength, mean velocity, and mean power during the bilateral standing press (BSP) in trained women.

**Methods:**

Sixteen trained women (age = 20.0 ± 2.30 years; body mass = 67.63 ± 6.68 kg; height = 1.65 ± 0.06 m) participated in this repeated-measures study. Absolute reliability was assessed using the coefficient of variation (CV), and relative reliability was assessed using the intraclass correlation coefficient (ICC) for a bilateral standing press (BSP) exercise protocol performed at intensities of 30% and 60% of isometric peak force (IPF).

**Results:**

Mean muscle strength showed acceptable absolute reliability and extremely high relative reliability at both 30% IPF (CV = 6.51%; ICC = 0.94) and 60% IPF (CV = 5.03%; ICC = 0.97). Mean velocity showed extremely high relative reliability at 60% IPF (ICC = 0.92), but its absolute reliability was not acceptable at either 30% IPF (CV = 10.10%; ICC = 0.75) or 60% IPF (CV = 14.16%; ICC = 0.92). Mean power also exceeded the predefined CV threshold at 30% IPF (CV = 11.16%; ICC = 0.85) and at 60% IPF (CV = 14.35%; ICC = 0.89).

**Conclusions:**

These findings suggest that the BSP protocol using functional electromechanical dynamometry is reliable for assessing mean muscle strength in trained women. However, mean velocity and mean power should be interpreted with caution, as their absolute reliability did not reach the predefined threshold of acceptable reliability.

## Introduction

In sports performance, there is a recent and growing interest in establishing standardized protocols and developing valid and reliable instruments to assess the muscular strength exerted by athletes in different exercises and natural movements (James et al., 2024; Knauer & Schroeder, 2025; García-Sánchez et al., 2024). The evaluation of conditioning capacities and protocols that reproduce the specific motor demands of each sport in training and competition has led to the consolidation of methodologies based on the objectivity, reproducibility, and stability of measurements (García-Sánchez et al., 2024; Mihaila et al., 2024; González-Ramírez et al., 2023; Fristrup et al., 2024). Advances in sports technology have enabled the highly accurate estimation of critical performance parameters when determining maximum strength, movement speed, and mechanical power applied to specific sporting actions (Morenas-Aguilar et al., 2025). Among the most innovative resources are inertial devices, mobile applications, and functional electromechanical dynamometry (FEMD), whose use optimizes the accuracy of evaluating various manifestations of strength. This has contributed to a greater understanding of the role of muscle quality in health prevention in older adults, injury treatment, and performance optimization (Chavda et al., 2024; Collings et al., 2025; Aerts et al., 2025; Norris et al., 2024; García-Ramos, Suzovic & Pérez-Castilla, 2019). Assessing limb strength in actions such as kicking, punching, or throwing requires precise and validated methods that allow for the quantification of different manifestations of muscle strength (García-Ramos, Suzovic & Pérez-Castilla, 2019; Kardor et al., 2023; Loepfe et al., 2025) in exercise protocols that closely resemble actual sporting actions (Morenas-Aguilar et al., 2025). Among the most widely used tools for evaluating muscle strength are isokinetic devices, which allow for the analysis of maximum torque, isolated muscle function, and the determination of balances between agonist and antagonist muscles, as well as the relationship between concentric and eccentric actions, providing relevant information for preventing functional imbalances and optimizing performance (Si et al., 2025; Spudić & Nosaka, 2025; Darbandi et al., 2025; Leahy, Florkiewicz & Shotwell, 2024). Conversely, FEMD has been used to assess isometric strength, maximal strength, and linear strength in various body segments, and has been associated with being a sensitive indicator of neuromuscular function (Del-Cuerpo et al., 2023; Mula-Pérez et al., 2024; Andrades-Ramírez et al., 2025). In throwing tests, which include velocity-controlled measurement, FEMD is used to assess the functional transfer of strength to sport-specific movements, such as the handball throw, integrating upper body power and intermuscular coordination (Zhang et al., 2024; Chirosa-Ríos et al., 2023; Cifuentes-Zapata et al., 2025).

The use of the FEMD is significant due to its ability to provide accurate and versatile measurements of maximal strength, maximum velocity, and maximum power under dynamic conditions for analytical and functional exercises (Andrades-Ramírez et al., 2024; Rodríguez-Perea et al., 2021). Previous studies on the reliability of the FEMD have demonstrated high relative and absolute reliability (ICC > 0.90), as well as low SEM values (Del-Cuerpo et al., 2023; Jerez-Mayorga et al., 2021; Baena-Raya et al., 2023; Sánchez-Sánchez et al., 2021), confirming its suitability for assessing maximal or peak isometric and dynamic strength in clinical and sports performance contexts (García-Buendía et al., 2024; Rodríguez-Perea et al., 2025; Morenas-Aguilar et al., 2024b, 2024a). The use of these devices to record continuous force-time data under sport-specific conditions and for different types of contractions (isometric, concentric, eccentric, and elastic) makes them a valid and reliable alternative to traditional isokinetic or manual dynamometry protocols (Rodríguez-Perea et al., 2025; Morenas-Aguilar et al., 2024b, 2024a).

In all cases, the analyzed values were the peak values of force, velocity, and power. None of these studies analyzed the reliability of average values across sets and repetitions, which could be of great interest when it is necessary to analyze not only peak values but also the average values of force, velocity, and power in a single repetition or set of exercises.

Therefore, the purpose of this study was to analyze the absolute and relative reliability of a functional electromechanical dynamometer (FEMD) for determining mean muscle strength, mean velocity, and mean power during a bilateral standing press (BSP) exercise at 30% and 60% of isometric peak force (IPF) in trained women.

## Materials and Methods

### Study design

A repeated-measures design was used to determine the reliability of the FEMD for mean force (MF), mean velocity (MV), and mean power (MP) during a BSP exercise. Each test was performed at the Physical Activity Sciences Laboratory of the University of the Americas, Santiago, Chile. Tests were conducted at the same time of day (±30 min) for each participant and under standardized temperature (≈21 °C) and humidity (≈60%) conditions. Familiarization sessions on the use of the FEMD and BSP were conducted before the assessments at 30% and 60% of individual IPF.

### Participants

Sixteen female participants (age = 20.0 ± 2.30 years; body mass = 67.63 ± 6.68 kg; and height = 1.65 ± 0.06 m) participated in the study. All participants had at least 3 years of strength training experience, maintained a training frequency of four to six sessions per week, and had no physical limitations or reported upper-limb injuries in the previous 6 months. The study was approved by the Ethics Committee of the University of Granada (Research Ethics Committee approval: 3074/CEIH/2022) and complied with the Declaration of Helsinki (World Medical Association, 2013). All participants provided written informed consent to participate in the study.

### Instruments

To determine body composition variables, the same recommendations as in a previous study (Cifuentes-Zapata et al., 2025) were followed. Body mass (kg) was assessed using a digital scale (Model InnerScan BC-554^®^; TANITA, Tokyo, Japan), and height (m) was determined using a stadiometer (Model 700^®^; SECA, Hamburg, Germany). Body mass index (BMI) was calculated by dividing body mass in kilograms (kg) by height in meters squared (m^2^).

Mean muscle strength, mean velocity, and mean power were assessed using a FEMD (Model Research; Dynasystem, Granada, Spain) with a precision of 3 mm for displacement, 100 g for detected load, a sampling frequency of 1,000 Hz, and a velocity range of 0.05 to 2.80 m · s^−1^. Displacement and velocity data were collected using a 2,500 ppr encoder connected to the roller. A load cell analyzed the stress applied to the recording system using an analog-to-digital converter with 12-bit resolution.

### Familiarization protocol

Prior to data collection, participants attended three 45-min sessions to familiarize themselves with the assessment procedure, the BSP execution protocol, and the use of the FEMD. In all sessions, four sets of ten repetitions of the BSP were performed with a load of 15% of body mass. This fixed familiarization load was selected to prioritize technical learning, movement standardization, and participant safety before exposing participants to individualized IPF-based testing loads. Instructions were always the same and were given by a single member of the research team; feedback was provided only during the familiarization phase.

Before each session, all participants performed a general warm-up consisting of 5 to 6 min of low-intensity treadmill running (heart rate of 130 beats per minute, measured with a Polar M400) and 5 min of upper and lower limb joint mobility exercises. The specific warm-up consisted of three sets of five repetitions with a 5 kg barbell, followed by three additional repetitions in the FEMD free mode using a simple pulley and grip system. During the final familiarization session, isometric peak force (IPF) was assessed during the BSP exercise (Cifuentes-Zapata et al., 2025; Cifuentes-Zapata et al., 2024) (Fig. 1A).

**Figure 1:**
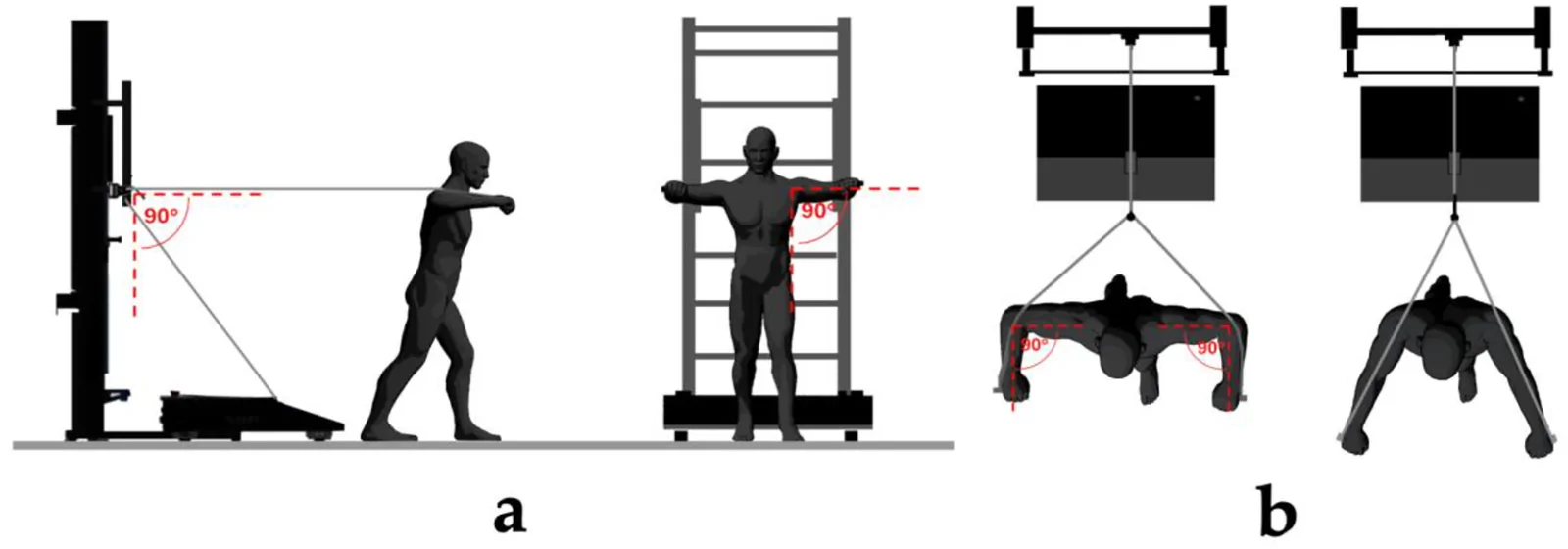
(A) Isometric peak force assessment in the bilateral standing press position. (B) Bilateral standing press exercises up to maximum elbow extension for intensities of 30% and 60%.

### Assessment protocol

After the familiarization sessions and IPF assessment, mean force, mean velocity, and mean power were measured at intensities of 30% and 60% of individual IPF. The IPF value obtained during the isometric test was used to calculate the external load applied by the FEMD for each participant; specifically, the device was configured in dynamic mode with a resistance equivalent to 30% or 60% of the participant-specific IPF value, expressed in kg according to the FEMD software output. To control for learning and order effects, the initial testing intensity was randomized. For each intensity condition, participants performed three sets of five repetitions, with 2 min of passive rest between sets and 5 min between intensity conditions. The BSP exercise was performed standing, with the dominant foot projected 20–30 cm forward, knees extended, and the non-dominant leg in 15–20° hip extension. Participants were instructed to avoid pelvic anteversion (0° tilt from vertical) to maintain trunk position. The handle was gripped with the wrist pronated, elbows flexed to 90°, and the shoulder abducted to 90° relative to the vertical. Maximal elbow extension and shoulder adduction were then performed as rapidly as possible during all repetitions and sets, as shown in Fig. 1B. Visual feedback was provided during all repetitions. Figure 1 shows the exercise sequence for IPF assessment and the dynamic BSP exercise. The results were exported from the device to a digital spreadsheet for further analysis. For the analysis, the mean values of MF, MV, and MP were calculated across valid repetitions and sets for each condition.

### Statistical analysis

Means and standard deviations (SD) were used to report descriptive statistics. The normality of the data distribution was analyzed using the Shapiro-Wilk test. Outliers were inspected before reliability analysis using graphical procedures and standardized values. Observations exceeding ±3 SD were considered potential outliers and were checked against the raw records for technical or execution errors; no valid observations were removed solely on statistical grounds. The paired-samples t-test and repeated-samples effect size (ES) were used to compare mean force, mean velocity, and mean power between the two sessions. The criteria for interpreting the magnitude of the ES were as follows: very large (>2.00), large (1.20–2.00), moderate (0.60–1.19), small (0.2–0.59), and trivial (<0.20) (Hopkins, 2015). Absolute reliability was measured using the coefficient of variation (CV), and relative reliability was assessed using the intraclass correlation coefficient (ICC). The same testing conditions and device were used in both sessions. A two-way mixed-effects, consistency, single-measurement intraclass correlation coefficient model [ICC(3,1)] was applied, following the Shrout and Fleiss convention described by Koo & Li (2016). High reliability (CV ≤ 5%) and acceptable reliability (CV ≤ 10%) were considered (Weir, 2005). The ICC was interpreted as follows: ≥0.90 = extremely high, 0.70–0.89 = high, 0.50–0.69 = moderate, 0.30–0.49 = low, and <0.30 = very low reliability (Brady, Harrison & Comyns, 2020; Koo & Li, 2016). Bland-Altman plots were calculated to show systematic bias and 95% limits of agreement between test and retest (Boehringer & Whyte, 2019). Heteroscedasticity of errors in Bland-Altman plots was defined as a coefficient of determination (R^2^) > 0.1 (Atkinson & Nevill, 1998). A 95% confidence interval was used in all cases, and statistical significance was defined as *p* < 0.05. All reliability analyses were performed using a custom spreadsheet (Hopkins, 2015), while other statistical analyses were performed using JASP software (version 0.16.4).

## Results

No significant test-retest differences were observed for MF, MV, or MP at either intensity (*p* > 0.05). The ES values were trivial for all variables and conditions (ES = 0.00–0.19), including MF at 30% IPF (ES = 0.00) and MF at 60% IPF (ES = 0.01). In the test–retest reliability analysis, MF showed acceptable absolute reliability at 30% IPF (CV = 6.51%) and 60% IPF (CV = 5.03%), together with extremely high relative reliability at both intensities (ICC = 0.94 and 0.97, respectively). MV at 60% IPF showed extremely high relative reliability (ICC = 0.92), but its CV was above the predefined threshold for acceptable absolute reliability (CV = 14.16%). MV at 30% IPF (CV = 10.10%; ICC = 0.75), MP at 30% IPF (CV = 11.16%; ICC = 0.85), and MP at 60% IPF (CV = 14.35%; ICC = 0.89) also exceeded the CV threshold. Therefore, ICC and CV were interpreted as complementary indices: high ICC values indicated ranking consistency across participants, whereas CV values >10% indicated limited absolute reliability for monitoring individual change in MV and MP (Table 1).

**Table 1:** Test–retest reliability of mean force, mean velocity, and mean power during the bilateral standing press.

	Mean ± SD		*p*-value	ES	CV%	ICC
	Test	Retest			(95% CI)	(95% CI)
Mean force (kg)						
30% IPF	9.20 ± 1.31	9.20 ± 0.97	0.99	0.00	6.51 [4.81–10.07]	0.94 [0.82–0.98]
60% IPF	19.17 ± 3.10	19.25 ± 2.42	0.89	0.01	5.03 [3.72–7.79]	0.97 [0.91–0.99]
Mean velocity (m · s^−1^)						
30% IPF	1.15 ± 0.17	1.18 ± 0.17	0.47	0.13	10.10 [7.46–15.63]	0.75 [0.43–0.91]
60% IPF	0.70 ± 0.28	0.76 ± 0.25	0.14	0.17	14.16 [10.46–21.91]	0.92 [0.79–0.97]
Mean power (W)						
30% IPF	112.11 ± 16.49	113.36 ± 14.52	0.77	0.04	11.16 [7.67–20.37]	0.85 [0.50–0.94]
60% IPF	140.62 ± 58.66	152.22 ± 50.12	0.13	0.19	14.35 [10.60–22.21]	0.89 [0.72–0.96]

**Note:**

m · s^−1^, meters per second; *p*-value, significance level; SD, standard deviation; ES, Cohen’s d effect size; CV%, coefficient of variation expressed as a percentage; ICC, intraclass correlation coefficient; IPF, isometric peak force; 95% CI, 95% confidence interval.

Bland-Altman analyses showed small systematic bias for MF at 30% IPF (bias = −0.001 kg; LoA = −1.660 to 1.658 kg), MF at 60% IPF (bias = 0.045 kg; LoA = −2.632 to 2.722 kg), MV at 30% IPF (bias = 0.030 m · s^−1^; LoA = −0.296 to 0.356 m · s^−1^), MV at 60% IPF (bias = 0.057 m · s^−1^; LoA = −0.230 to 0.343 m · s^−1^), MP at 30% IPF (bias = 1.258 W; LoA = −32.73 to 35.25 W), and MP at 60% IPF (bias = 11.60 W; LoA = −46.65 to 69.84 W). Heteroscedasticity was observed for MF at 30% IPF (R^2^ = 0.183) and MF at 60% IPF (R^2^ = 0.258), indicating that the magnitude of the error may increase across the measurement range for the force outcomes. The remaining outcomes showed R^2^ values below the predefined threshold (MV 30% IPF = 0.003; MV 60% IPF = 0.053; MP 30% IPF = 0.018; MP 60% IPF = 0.006), as shown in Fig. 2 and Table 2.

**Figure 2:**
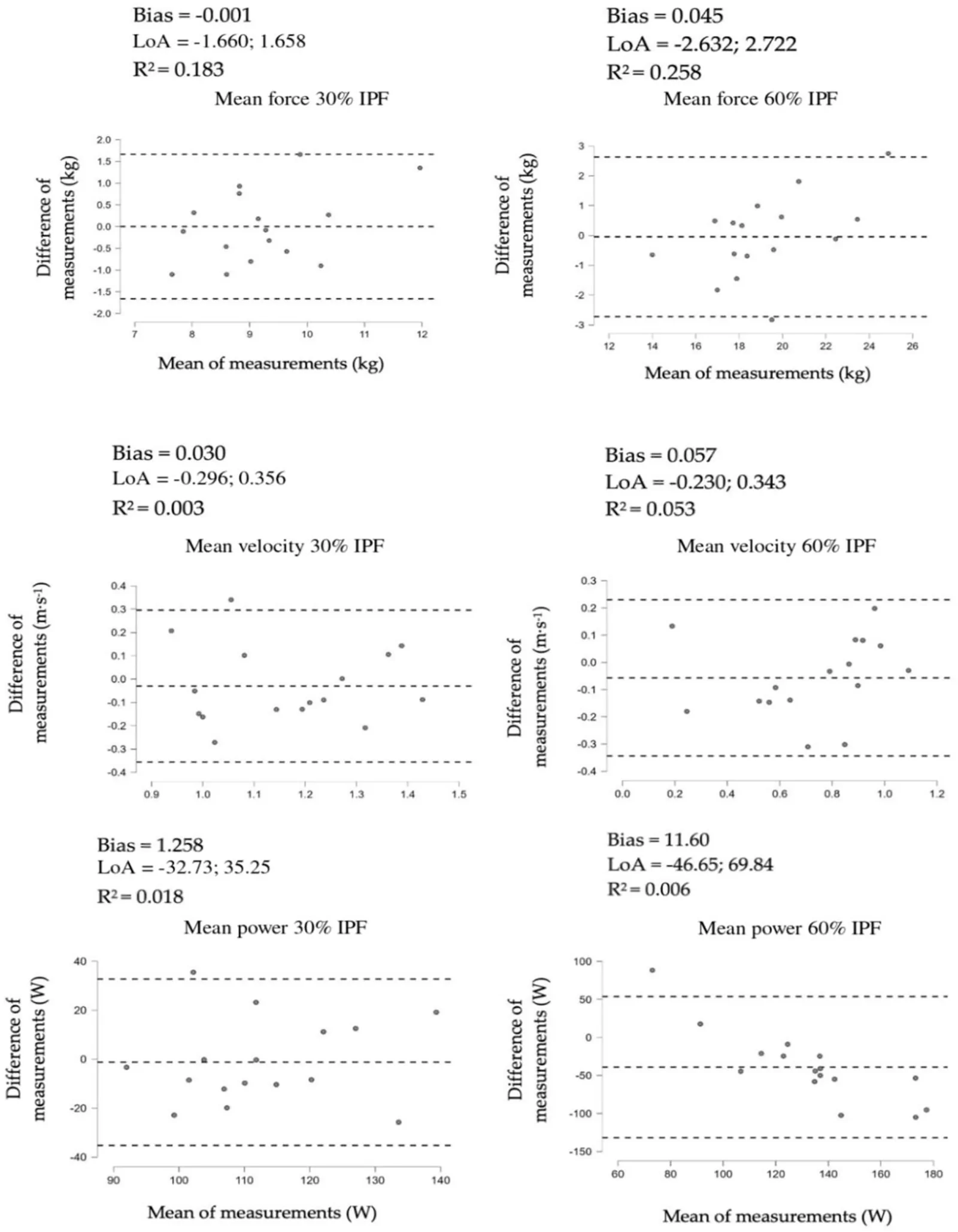
Bland-Altman analysis. IPF, isometric peak force; LoA, limits of agreement.

**Table 2:** Bland-Altman systematic bias, limits of agreement, and heteroscedasticity analysis for each outcome.

Variable	Intensity	Bias	95% LoA	R^2^
Mean force	30% IPF	−0.001 kg	−1.660 to 1.658 kg	0.183
Mean force	60% IPF	0.045 kg	−2.632 to 2.722 kg	0.258
Mean velocity	30% IPF	0.030 m · s^−1^	−0.296 to 0.356 m · s^−1^	0.003
Mean velocity	60% IPF	0.057 m · s^−1^	−0.230 to 0.343 m · s^−1^	0.053
Mean power	30% IPF	1.258 W	−32.73 to 35.25 W	0.018
Mean power	60% IPF	11.60 W	−46.65 to 69.84 W	0.006

**Note:**

LoA, limits of agreement; IPF, isometric peak force. Heteroscedasticity was defined as R^2^ > 0.10.

## Discussion

The aim of this study was to analyze the absolute and relative reliability of a functional electromechanical dynamometer (FEMD) for determining mean muscle strength, mean velocity, and mean power during a bilateral standing press (BSP) exercise in trained women. The main findings were that MF showed acceptable absolute reliability and extremely high relative reliability at both 30% and 60% IPF, whereas MV and MP showed less favorable absolute reliability despite moderate-to-high relative reliability estimates. The absence of significant test-retest differences and the trivial ES values suggest no systematic learning or fatigue effect between sessions; however, the interpretation of reliability must consider both ICC and CV because these indices address different aspects of measurement consistency (Warneke et al., 2025).

For mean force, the present findings support the use of FEMD during the BSP when the objective is to monitor average upper-limb force output in trained women. The CV values for MF remained within the acceptable threshold at both intensities, and the ICC values were extremely high. These results are consistent with previous FEMD studies showing high relative and absolute reliability when joint position, range of motion, and familiarization are standardized (Martínez-García et al., 2020; Del-Cuerpo et al., 2023; García-Buendía et al., 2024). Therefore, MF appears to be the most robust outcome of the current BSP protocol for applied monitoring and longitudinal assessment.

For mean velocity, the interpretation was more cautious. Although MV at 60% IPF reached an ICC of 0.92, its CV was 14.16%, which exceeded the predefined threshold for acceptable absolute reliability. This finding indicates that participants tended to maintain their relative ranking across sessions, but the within-participant error was too large for confident individual-level monitoring (Jukic et al., 2023). The lower reliability observed at 30% IPF may be related to the greater technical and neuromuscular variability typically observed at lighter loads, where small changes in movement trajectory, range of motion, and intention to move rapidly can strongly affect velocity outputs (Martínez-Cava et al., 2020; Grgic et al., 2020; Thompson et al., 2020; Lecocq et al., 2025; Montoro-Bombú et al., 2025).

For mean power, the CV values also exceeded the acceptable threshold at both intensities, and the lower bounds of the ICC confidence intervals indicated imprecision. This is expected because power is derived from both force and velocity and therefore accumulates error from several sources, including external load configuration, range of motion, and movement trajectory (Pérez-Castilla et al., 2019; Weakley et al., 2021a, 2021b; González-Galán et al., 2024; Ruiz-Alias et al., 2024). Consequently, MP should not be prioritized as the primary monitoring variable in the current BSP protocol unless additional standardization and replication studies confirm acceptable absolute reliability.

The Bland-Altman analysis further clarified the practical interpretation of the results. Systematic bias was small for most outcomes, but heteroscedasticity was detected for MF at both 30% and 60% IPF, suggesting that the magnitude of the error may vary across the measurement range. In contrast, MV and MP did not show heteroscedastic patterns based on the predefined R^2^ criterion, although their wider CV values still limit their use for detecting small individual changes. These findings reinforce the need to report ICC and CV as complementary indices and to interpret reliability estimates alongside confidence intervals and limits of agreement.

The small sample size should also be considered when interpreting the findings. Although the study provides novel data in a specific and underrepresented population, the confidence intervals for MV and MP were wide, particularly at 30% IPF. Therefore, the reliability estimates for these variables should be interpreted as preliminary and should be replicated in larger samples. Future studies should also explore whether additional indices of measurement error, such as mean absolute error or mean absolute percentage error, improve interpretability for practitioners using FEMD-derived variables in applied settings.

## Conclusions

Our results indicate that the FEMD-based BSP protocol at intensities of 30% and 60% IPF achieved acceptable absolute reliability and extremely high relative reliability for mean muscle strength. These results support the use of FEMD for monitoring and decision-making regarding upper-limb force-generating capacity in trained women. However, mean velocity and mean power did not reach acceptable absolute reliability according to the predefined CV threshold, despite some high ICC values; therefore, their interpretation should be approached with caution and accompanied by rigorous standardization of the assessment protocol and execution technique. These findings support FEMD as a reliable tool for assessing mean muscle strength during the BSP, while further methodological refinement is required to improve the reliability of mean velocity and mean power variables.

### Limitations

This study was conducted with a small sample of trained women and a single movement (bilateral standing press) at two relative intensities (30% and 60% of IPF), which limits the generalizability of the results. No *a priori* sample size calculation was performed (Mokkink et al., 2022); consequently, the confidence intervals for several ICC estimates, particularly MV and MP, were wide, and these outcomes should be interpreted with caution. The familiarization protocol used a fixed load based on body mass rather than individualized IPF, which may not have represented the subsequent testing intensities equally for all participants. The conclusions are also limited to a single FEMD device and laboratory conditions, without simultaneous validation against reference systems.

### Applicability and future research

The FEMD is a reliable device for assessing mean muscular force in upper-body performance monitoring and follow-up contexts in women with sports experience. Mean velocity and mean power should be interpreted with caution for individual monitoring until further studies confirm lower absolute measurement error. Future research should include larger samples, additional exercises, individualized familiarization loads, complementary indices of measurement error, and multicentre or field-based designs.

## Supplemental Information

10.7717/peerj.21777/supp-1 Supplemental Information 1 STROBE checklist.

10.7717/peerj.21777/supp-2 Supplemental Information 2 Raw Data. Database for peer review analysis

## Additional Information and Declarations

### Data Availability

The following information was supplied regarding data availability:

The raw data is available in the Supplemental Files.
